# Retinoic acid synthesis by NG2 expressing cells promotes a permissive environment for axonal outgrowth

**DOI:** 10.1016/j.nbd.2017.12.016

**Published:** 2018-03

**Authors:** Maria B. Goncalves, Yue Wu, Diogo Trigo, Earl Clarke, Tony Malmqvist, John Grist, Carl Hobbs, Thomas P. Carlstedt, Jonathan P.T. Corcoran

**Affiliations:** The Wolfson Centre for Age-Related Diseases, King's College London, Guy's Campus, London SE1 1UL, United Kingdom

**Keywords:** Retinoic acid, Exosome, Axonal guidance

## Abstract

Stimulation of retinoic acid (RA) mediated signalling pathways following neural injury leads to regeneration in the adult nervous system and numerous studies have shown that the specific activation of the retinoic acid receptor β (RARβ) is required for this process. Here we identify a novel mechanism by which neuronal RARβ activation results in the endogenous synthesis of RA which is released in association with exosomes and acts as a positive cue to axonal/neurite outgrowth. Using an established rodent model of RARβ induced axonal regeneration, we show that neuronal RARβ activation upregulates the enzymes involved in RA synthesis in a cell specific manner; alcohol dehydrogenase7 (ADH7) in neurons and aldehyde dehydrogenase 2 (Raldh2) in NG2 expressing cells (NG2 + cells). These release RA in association with exosomes providing a permissive substrate to neurite outgrowth. Conversely, deletion of Raldh2 in the NG2 + cells in our in vivo regeneration model is sufficient to compromise axonal outgrowth. This hitherto unidentified RA paracrine signalling is required for axonal/neurite outgrowth and is initiated by the activation of neuronal RARβ signalling.

## Highlights

• Raldh2, the enzyme for retinoic acid synthesis, is upregulated in NG2 + cells during axonal regeneration.
• Deletion of Raldh2 in NG2 + cells prevents regeneration.
• RA signalling modulates axonal pathfinding.
• Fine-tuned regulation of RA distribution via exosome transport

## 1 Introduction

The importance of RARβ signalling in adult CNS regeneration has been well documented (Agudo et al., 2010, Corcoran et al., 2000, Dmetrichuk et al., 2005, Koriyama et al., 2013, Puttagunta and Di Giovanni, 2011, Wong et al., 2006, Yip et al., 2006) and we have shown in our previous work that treatment with an RARβ agonist leads to axonal regeneration and functional recovery in a rat model of sensory root avulsion (Goncalves et al., 2015). An important aspect for axonal regeneration in the adult is appropriate axonal guidance since in the absence of permissive cues, the growing axons display haphazard growth and fail to reform functional connections even when the obstacles to regeneration are cleared (Harel and Strittmatter, 2006). RA, the endogenous ligand to the RARs, has been shown to act as a guidance cue: in vitro, neurites follow an RA gradient (Maden et al., 1998) and in retinoid deficient quails axonogenesis is impaired and the axons that do form present pathway deficits (Maden et al., 1996). The endogenous synthesis of RA requires sequential oxidative steps. Alcohol dehydrogenase 7 (ADH7) is the most abundant class IV ADH in the adult rodent CNS (Martinez et al., 2001) and oxidises retinol to retinal which is then converted into RA by Raldh2 (Niederreither et al., 1999). A type of glial cells expressing the nerve glial antigen 2 (NG2 + cells) participate in the endogenous RA signalling as they express Raldh2 (Kern et al., 2007). These cells populate the lesion site after spinal cord injury (SCI) and a temporal up-regulation of Raldh2 after injury has been previously described but the actual synthesis and function of RA has never been reported (Mey et al., 2005). The overall effect of NG2 + cells in axonal regeneration seems to depend largely on their phenotype which can yield dichotomic responses. Some studies suggest that NG2 + cells can be detrimental to axonal regeneration (Dou and Levine, 1994, Fidler et al., 1999, Jones et al., 2002, Tang et al., 2003, Ughrin et al., 2003), whereas numerous findings cast doubt on the general validity of this view (Busch et al., 2010, Jones et al., 2003, McTigue et al., 2006, Yang et al., 2006) and NG2 + cells have been shown to promote neurite outgrowth and to form, both in vitro and in vivo, extensive contacts with the growth cone (Yang et al., 2006).

Here we hypothesized that RA synthetized by NG2 + cells might be important for axonal guidance in the adult following neuronal injury. The animal model we chose for our experimental set up was a rat model of cervical sensory root avulsion due to its clinical translatability and the extent of the neural damage inflicted. In brachial plexus avulsion injuries the spinal root is avulsed at the interface between the central and peripheral nervous system (CNS and PNS). This results not only in the disconnection of the root from the cord but also in a longitudinal SCI (Hoeber, 2015). If left untreated, the affected spinal cord segments can deteriorate over about 1 month, with disintegration of neuronal networks and death of motor, sensory, and autonomic nerve cells. The clinical effect of such lesion is loss of motor, sensory and autonomic function.

Using an RARβ agonist to induce regeneration in the rat model of cervical dorsal root avulsion, as we had previously demonstrated (Goncalves et al., 2015), we found that axons grow preferentially towards RA synthetizing NG2 + cells. We show that RA is secreted in or in association with exosomes, and this controlled secretion of RA acts as an important axonal growth stimulating cue. The endogenous synthesis of RA is initiated by the activation of the RARβ signalling in neurons which results in the upregulation of the enzymatic machinery involved in RA biosynthesis. We validate the requirement for RA in our regeneration system by a Raldh2 siRNA lentiviral transfection study where the functional loss of the enzyme prevents the lesioned axons growth through the DREZ into the SC, even in the presence of RARβ agonist treatment. In this study, we have identified a novel role of the RA signalling in NG2 + cells-neuron communication during adult axonal regeneration.

## 2 Results

### 2.1 NG2 + cells form a bridge through the DREZ for regenerating axons which is dependent on RARβ upregulation and activation at the axons

**Fig. 1:**
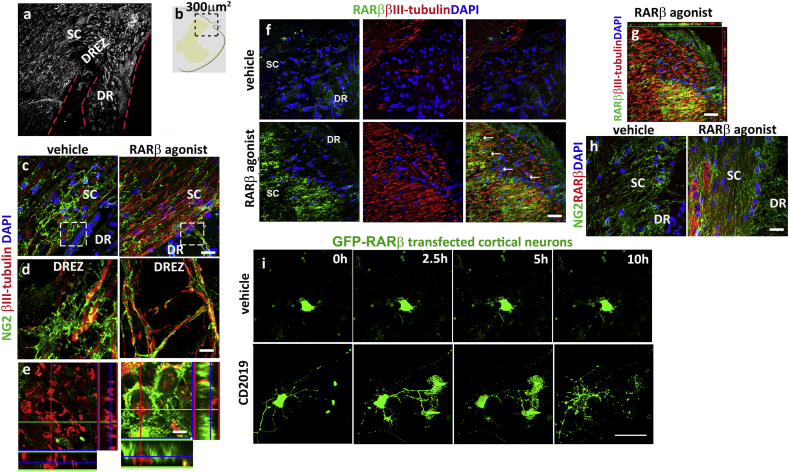
RARβ activation leads to NG2 + cells wrapping around regenerating axons and RARβ up-regulation at the axonal tip. (a) Representative image of the morphological areas studied: Dorsal root entry zone (DREZ) and adjacent spinal cord (SC) and dorsal roots (DR) of the injured rats with the pia boundary delineated in red. (b) Schematic representation of the area used for imaging and quantifications. (c) Expression of NG2 + cells in vehicle and RARβ agonist treated avulsed rats at the PNS-CNS interphase. Scale bar is 100 μm. (d) Higher magnification images of the insets delineated in (a) showing the morphological differences of NG2 + cells and the axonal tips in the two groups: NG2 + cells wrap around the axons at the DREZ in the regenerating SC whereas in vehicle treated rats, axonal tips are seen surrounded by arborized NG2 + cells processes. Scale bar is 30 μm. (e) Images obtained from different sections from the same treatment groups, showing a transversal plane of the positioning of the NG2 + cells and the axons at the DREZ. Scale bar is 15 μm. (f and g) RARβ is up-regulated in the axons at the dorsal horn in the RARβ agonist treated animals but absent in the NG2 + cells (h). (i) Live cell imaging of RARβ2 transfected cortical neurons over 10 hour post-treatment. Scale bar is 100 μm.

Using a rat model of cervical sensory root avulsion in which treatment with an RARβ agonist leads to axonal regeneration (Goncalves et al., 2015), we first looked at the expression pattern of NG2 + cells at the dorsal root entry zone (DREZ) comprising an area of 300 μm^2^ around the PNS-CNS interface (Fig. 1a and b). In vehicle treated rats, which do not regenerate (Goncalves et al., 2015), NG2 + cells bulged with multiple processes “docking” along various points of the axonal bundles (Fig. 1c–e). This phenotype has been ascribed to NG2 + cells with an anti-regeneration effect (Cregg et al., 2014, Vadivelu et al., 2015) and here is visualised by the blunted axonal tips adjacent to the NG2 + cell processes (Fig. 1d). In contrast, in RARβ agonist treated rats NG2 + cells exhibited long processes enwrapped around the axons that project into the DREZ (Fig. 1c–e).

To determine if this could be a direct effect of RARβ activation, we examined the pattern of expression of RARβ around the DREZ and found that in the RARβ agonist treated rats only, the receptor was highly abundant in the dorsal horn regenerating axons (see arrows in Fig. 1f, and h) but was absent in NG2 + cells, thus precluding a direct action of the agonist (Fig. 1f, h). To confirm the RARβ movement into the growing tip we transfected cortical neurons with a GFP-tagged RARβ2 expression construct, and using live cell imaging, monitored the movement of the receptor over 10 h, in the presence of vehicle or a specific RARβ agonist (CD2019). The RARβ agonist induced a remarkable increase of RARβ2 at the growing neurite tips that was evident after 2.5 h and peaked at 5 h of treatment (Fig. 1i). This effect was not seen in the vehicle treated transfected cells (Fig. 1i).

### 2.2 In neuron-NG2 + cell cultures NG2 + cells can promote neurite outgrowth in the presence of retinol and retinal

**Fig. 2:**
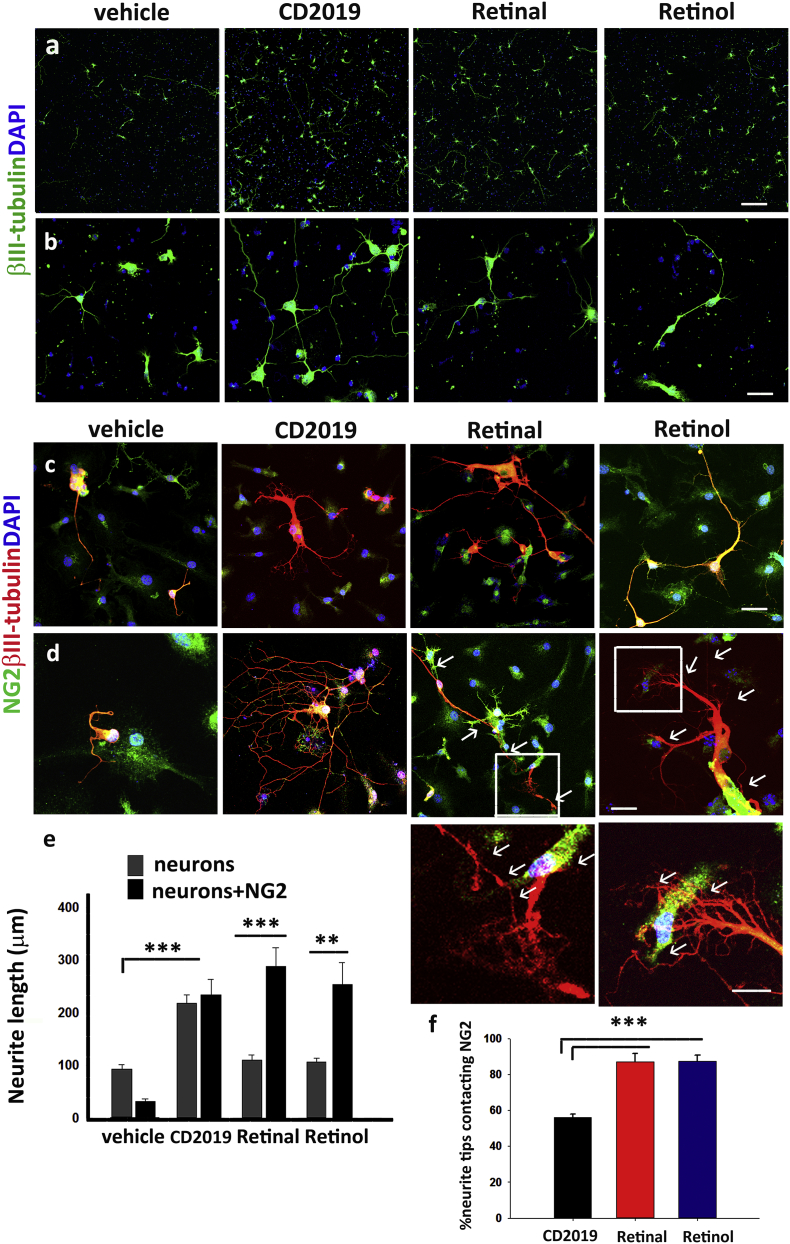
Effect of RA precursors and a RARβ agonist in neurite outgrowth in neuron and neuron-NG2 cultures. (a and b) CD2019 induces neurite outgrowth in cortical neuron cultures but not retinal nor retinol. Upper panel shows lower magnification images (a), scale bar is 300 μm and lower panel (b) higher magnification pictures of the same cultures, scale bar is 50 μm. (c–d) CD2019, retinal and retinol increase neurite length in neuron-NG2 + cells cultures. The upper panel shows lower magnification images, scale bar is 100 μm (c) and the lower panel (d) shows images of the same cultures in higher magnification, scale bar is 50 μm. The white arrows indicate the points of physical contact between the NG2 + cells and neurites. (e) Quantification of the length of the longest neurites per culture condition obtained from six random fields and done in triplicate per culture condition. (f) NG2 + cells attract and guide growing neurite in the presence of retinal and retinol: there is a significant increase in the % of growing neurites that contact with the NG2 + cells, illustrated by the white arrows in these photomicrographs. Scale bar is 30 μm. Data represent Mean ± SEM. **p ≤ 0.01***p ≤ 0.001, one-way ANOVA followed by Tukey's test.

To dissect out the RA signalling between neurons and NG2 + cells we cultured mouse cortical neurons alone or with mouse NG2 + cells and treated the cultures with vehicle or CD2019, or the RA precursors, retinal and retinol. We chose to use this type of neurons since they are easier to grow and the growth response to NG2 + cells and to retinoids has been shown to be translatable between sensory and CNS neurons (Agudo et al., 2010, Corcoran et al., 2000, Filous et al., 2014, So et al., 2006). After 3 days, neurite length was measured and we found that CD2019, retinal and retinol, all induced a significant increase in neurite growth (~ 2-fold, *p* ≤ 0.001; ~ 3-fold, *p* ≤ 0.001 and ~ 2.5-fold, *p* ≤ 0.01 respectively compared to vehicle), in the co-cultures but with different growth patterns (Fig. 2a–e). CD2019 resulted in the random extension of neurites often seen going around in loops (Fig. 2c and d). In striking contrast, both the retinol and retinal induced a stream of neurites towards and beyond the NG2 + cells with significantly higher points of contact (33.3%, *p* ≤ 0.001) between neurite tips and NG2 + cells than the CD2019 treated co-cultures (Fig. 2f). This is highly suggestive of a pathfinding effect that was absent with the CD2019 treatment. However, the RARβ agonist, as had been shown before (Agudo et al., 2010) was the only treatment that induced neurite growth when the neurons were cultured without the NG2 + cells (Fig. 2a, b and e), indicating that activation of RARβ is required for neurite growth. This suggests that synthesis of RA takes place in retinal and retinol treated NG2 + cell-neuron co-cultures which then activates RARβ in the neurites.

### 2.3 RARβ signalling initiates a neuron-NG2 + cell endogenous RA synthesis conducive to axonal outgrowth

**Fig. 3:**
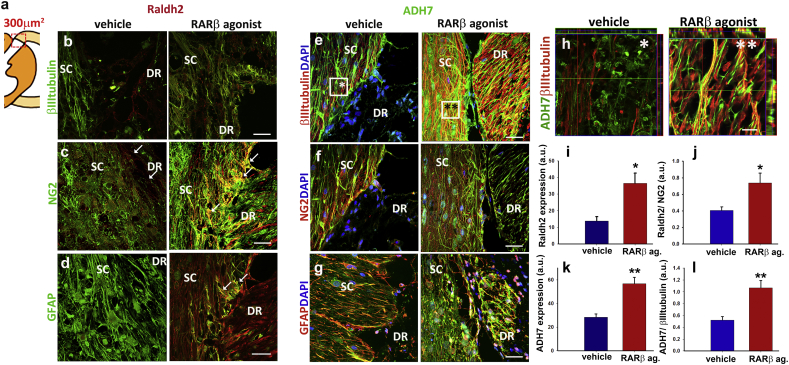
Expression of Raldh2 and ADH7 in axonal tracts, NG2 + cells and astrocytes at the PNS-CNS interface. (a) Schematic representation of the tissue analysed with the area used for quantitative analyses shown in the red box (300 μm^2^). (b) Immunostaining for Raldh2 and βIII-tubulin shows absence of Raldh2 in axons in the DR and SC of vehicle and RARβ agonist treated avulsed rats. Scale bars is 100 μm. (c) Raldh2 is highly upregulated at the DREZ in the RARβ agonist treated rats but absent in the vehicle group (see arrows). Scale bars is 100 μm. (d) Immunostaining for Raldh2 and GFAP shows that Raldh2 is present in some of the astrocytes at the DREZ in the RARβ treated group only (see arrows). Scale bars is 100 μm. (e) ADH 7 is upregulated only in axons of RARβ agonist treated rats in both the DR and the SC. Scale bars is 100 μm. (f) NG2 + cells express very little if any ADH7 in both animal groups. Scale bars is 100 μm. (g) ADH7 is present in very few astrocytes in vehicle and RARβ agonist treated rats. Scale bars is 100 μm. (h) High magnification of Z-stack images of the dorsal horn (indicated boxed in squares with * for vehicle and ** for RARβ agonist treated), show that ADH7 is completely absent from axons in vehicle treated group but in contrast is present in the axons of the RARβ agonist treated rats. Scale bar is 30 μm. (i) Quantification of Raldh2 expression at the interface between DR and SC, expressed in means of pixels of fluorescence [arbitrary units (a.u.)], and of (j) Raldh2 in NG2 + cells in the same area. (k) Similar quantification of ADH7 and (l) ADH7 in axons in equivalent zones of the PNS-CNS interface. Data represent Mean ± SEM. *p ≤ 0.05, **p ≤ 0.01, one-way ANOVA followed by Tukey's test.

To clarify the role of NG2 + cells and neurons in the endogenous RA synthesis we next looked at the expression of Raldh2 and ADH7 around the injury area in RARβ agonist treated rats compared to vehicle treated ones. A schematic representation of the areas analysed is shown in Fig. 3a. Raldh2 was absent from axons in both groups (Fig. 3b). In the RARβ agonist treated group only, Raldh2 was upregulated in NG2 + cells that formed tunnel-like cords at the PNS-CNS border and projected into the dorsal horn (Fig. 3c, arrows in mid panel). Quantitative analysis of Raldh2 expression demonstrates that it is significantly higher (65%, *p* ≤ 0.05 total expression and ~ 50% higher in NG2 + cells, *p* ≤ 0.05) expressed in RARβ agonist treated rats in the NG2 + cells at the DREZ when compared to controls (Fig. 3i and j), suggesting that a localized synthesis or RA is important for axonal guidance of the growing tip into the SC during regeneration. Raldh2 was also present in a discrete population of GFAP cells at the DREZ (Fig. 3d). In contrast, ADH7 was remarkably upregulated (~ 50% total, *p* ≤ 0.05) in the axons of RARβ agonist treated rats (Fig. 3e, h, k and l) but absent in NG2 + cells and expressed at low levels in the astrocytes (Fig. 3f, g), suggesting the major source of retinal is neuronal in this regenerating system. The fact that both enzymes involved in the biosynthesis of RA are upregulated in the RARβ agonist treated animals suggests that despite the presence of the agonist, endogenous RA synthesis plays an important role in the regeneration process.

**Fig. 4:**
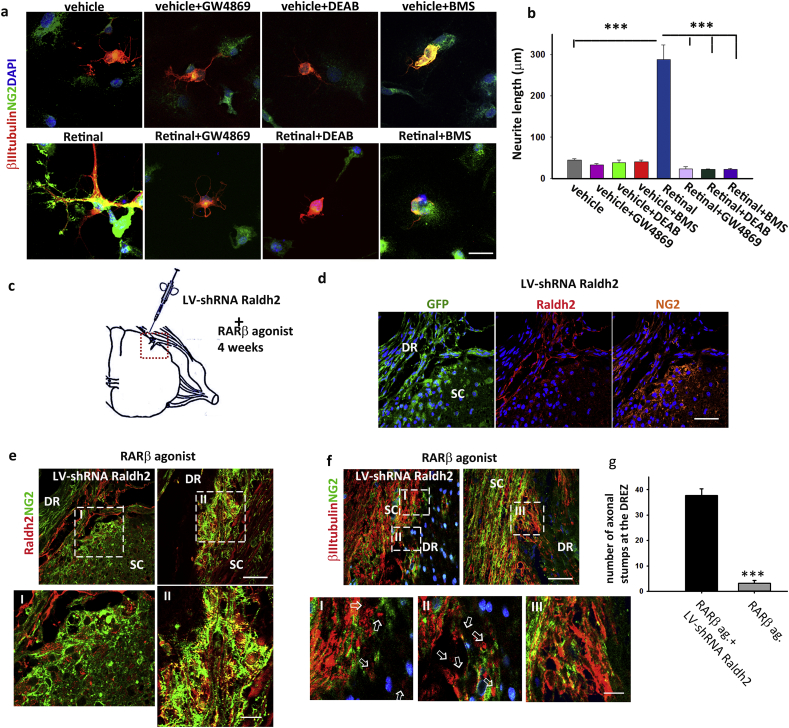
Compartmentalization of RA synthesis is essential for exosome mediate RA transfer in neurite outgrowth. (a) Neurite growth in neuron-NG2 + cell cultures treated for 72 h with vehicle and retinal, alone or with the addition of BMS, GW4869 or DEAB. Scale bar is 50 μm. (b) Quantification of the longest neurite lengths in the cultures showed a significant reduction in the retinal induced neurite outgrowth when: either activation of RARs is inhibited (BMS) or RA synthesis (DEAB) or exosome transfer are prevented (GW4869). Data represent mean ± SEM. **p ≤ 0.01***p ≤ 0.001, One-way ANOVA followed by Tukey's test. (c) Diagram illustrating LV shRNA Raldh2 injection site at the DREZ, performed at the same time of injury in C5-T1 of rats that were subsequently treated with the RARβ agonist for 4 weeks. (d) Expression of GFP, Raldh2 and NG2 in RARβ treated rats that received LV shRNA Raldh2, shows effective transduction. Scale bar is 100 μm. (e) Loss of Raldh2 in the NG2 + cells at the DREZ in the same section shown in (d) compared to the expression of Raldh2 in the NG2 + cells of a RARβ agonist treated group that had not been transfected. Scale bar is 100 μm. Insets (I) and (II) show a higher magnification image of SC areas the differences in Raldh2 in NG2 + cells can be clearly seen between the two groups. Scale bar is 40 μm. (f) In the LV shRNA Raldh2 transduced rats, the axons fail to enter the SC and numerous axonal stumps remain at the DREZ (indicated by the white arrows in the lower insets, I and II). In contrast, in the non-transduced RARβ agonist treated animals, bundles of axons are seen entering the DREZ surrounded by NG2 + cells (seen clearly in the lower inset III). Scale bar is 100 μm for upper panel and 40 μm for lower insets (I, II and III). (g) Quantification of the axonal stumps at the DREZ. Data represent mean ± SEM. ***p ≤ 0.001, Student's *t*-test.

The generation of RA gradients as guidance cues has been described before during patterning of the developing CNS (Maden, 2007) and internal organs, in the latter via a vesicular release of RA (Tanaka et al., 2005). Given the lipophilic properties of RA, we explored the possibility of a vesicle associated mode of RA transfer and hypothesized that the NG2 + cells, where the last step in the synthesis of RA takes place, could secrete RA in or associated with exosomes. Exosomes have a prominent role in fine-tuned cell-cell communication (Bang and Thum, 2012), and the controlled release of RA in a confined membrane structure would create a spatiotemporal source of RA to which the adjacent neuronal tips would be recipient to. To test the requirement for the synthesis of RA in promoting neurite growth, we treated NG2 + cell-neuron cultures with either vehicle or retinal alone or in the presence of DEAB, a Raldh2 inhibitor. We found that DEAB did not alter the length nor preference of the neurites towards the NG2 + cells compared to vehicle alone, but it abolished the neurite outgrowth effect of the retinal, suggesting that RA is essential in this process (Fig. 4a and b). We next treated the co-cultures with vehicle or retinal in the presence of a pan-RAR agonist, BMS189453, to assess the effect of blocking the RA signalling. This too resulted in the abrogation of neurite growth towards the NG2 + cells (Fig. 4a and b) confirming an RAR mediated response. Finally, to determine the importance of exosome transfer in the RA mediated neurite growth and guidance in the NG2 + cell-neuron cross-talk, we added GW4869, an exosome inhibitor, to the vehicle/retinal treated co-cultures. This resulted in the revocation of neurites growing towards the NG2 + cells and significantly diminished neurite length (Fig. 4a and b). This data taken together, demonstrates that RA extracellular transfer in exosomes is required for NG2 + glia induced neurite outgrowth.

Next, to directly assess in vivo the requirement of RA synthesis in axonal guidance, we injected a GFP tagged lentivirus with a short hairpin RNA to silence Raldh2 (LV shRNA Raldh2) into the DREZ at the time of injury and treated the rats for 4 weeks with the RARβ agonist as before (Fig. 4c). Immunohistochemistry confirmed the efficient transduction of the lentivirus (Fig. 4d). We then compared the expression of Raldh2 in NG2 + cells at the DREZ in rats that have been transduced and rats that have received the RARβ agonist treatment only. There was very little, if any, Raldh2 in the NG2 + cells in the lentivirus injected rats in contrast with the robust presence of the enzyme in non-transfected ones (Fig. 4e, and insets 4eI and 4eII).

To evaluate the role of Raldh2 in axonal regeneration through the PNS-CNS interface, we looked at the axons entering the SC since growth and guidance are indissociably required for appropriate axonal regeneration (Harel and Strittmatter, 2006). A typical feature of stalled axonal growth is the presence of dystrophic endings with various shapes and sizes that remain at the lesioned site (Silver and Miller, 2004). We found that in the absence of Raldh2 numerous dystrophic axonal tips (axonal stumps) were seen at the DREZ whereas in the non-transfected group, bundles of axons projected into the SC surrounded by NG2 + cells processes (Fig. 4f and insets 4fI, II and III). Quantification of the number of the axonal stumps (arrows in Fig. 4fI and II and g), confirmed that the synthesis of RA is essential for appropriate axonal guidance through the DREZ as there was a significant increase (~ 8-fold higher, *p* ≤ 0.001) of growth arrested axons in the LV-shRNA Raldh2 RARβ agonist treated transfected rats when compared to agonist treated non-transfected.

### 2.4 NG2 + cells are a source of RA which is associated with exosomes

**Fig. 5:**
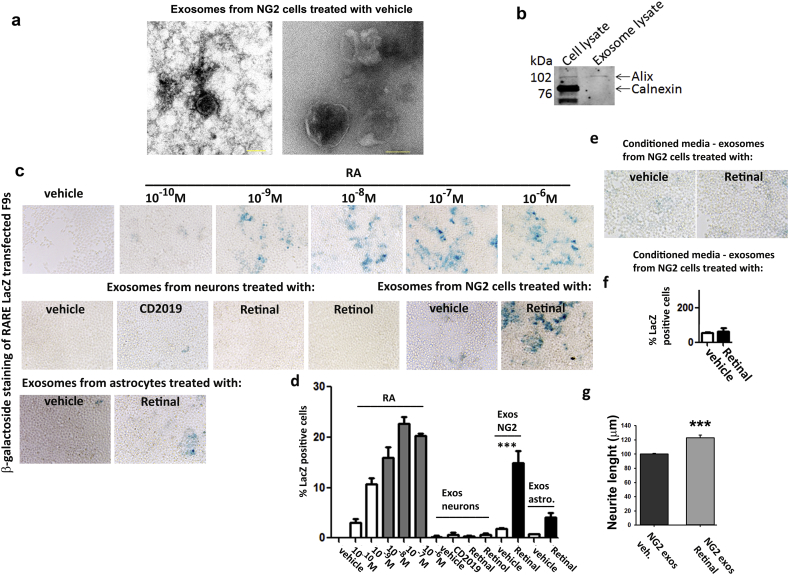
NG2 + cells are the main source of RA. (a) Exosomes isolated from NG2 + cells conditioned media detected with electron microscopy, scale bar is 50 nm and 100 nm (b) and by Western blotting. (c) β-galactoside staining of RARE LacZ transfected F9 cells showing RA in exosomes isolated from neuronal cultures treated with vehicle, CD2019, retinal or retinol, or exosomes from NG2 + cells or astrocyte cultures treated with vehicle or retinal. (d) Quantification of RA shows that NG2 + cells are the main source of RA., Data represent Mean ± SEM. ***p ≤ 0.001, one-way ANOVA, followed by Tukey's test. (e) β-galactoside staining of RARE LacZ transfected F9 cells showing RA in NG2 + cells conditioned media after exosome extraction. The culture conditioned were vehicle or retinal. (f) Quantification of RA shows that there is very little RA in the conditioned media after exosome extraction in either culture condition and no statistical significant difference between the two. Data represent Mean ± SEM. Student's *t*-test. (g) Significant increase in neurite outgrowth of neurons cultured with exosomes from NG2 + cells that had been cultured with retinal but not with exosomes from NG2 + cells treated with vehicle. Data represent Mean ± SEM. ***p ≤ 0.001, Student's *t*-test.

**Fig. 6:**
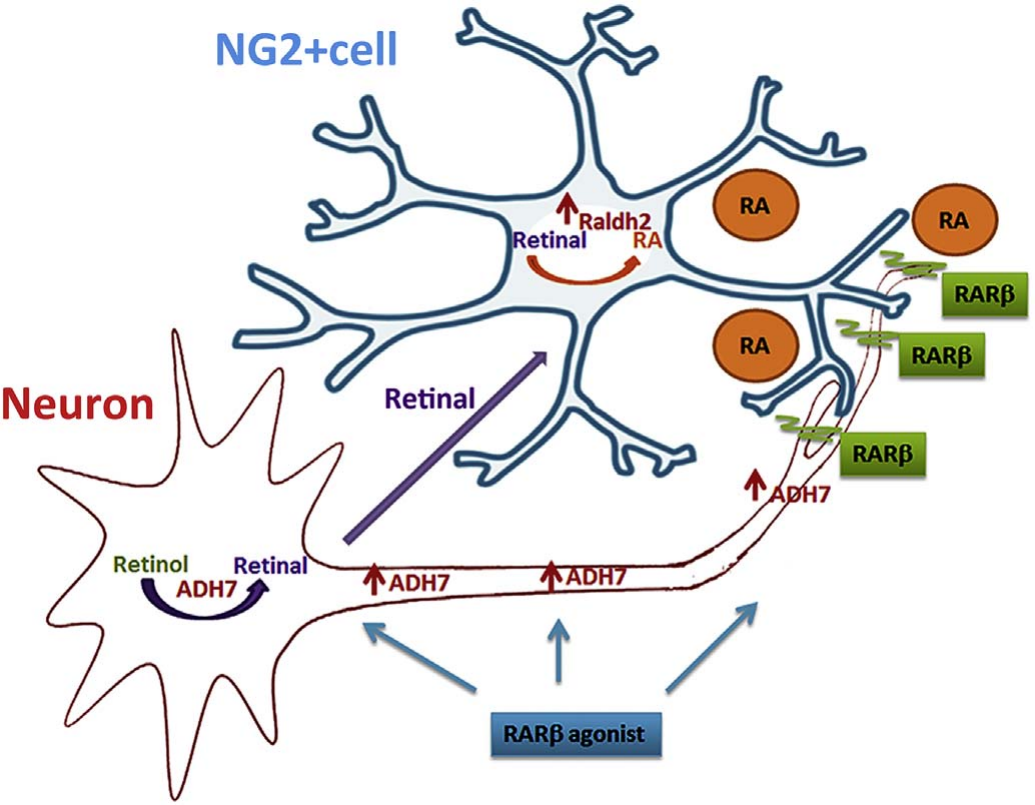
Neuron-NG2 + cell RA synthesis and transfer in association with exosomes that is induced by neuronal activation of RARβ agonist. Schematic representation of a novel signalling pathway in axonal/neurite outgrowth. Treatment with an RARβ agonist induces upregulation of the receptor at the axonal tips and this promotes the endogenous synthesis of RA, via upregulation of ADH7 in the neuron. This increases the synthesis of retinal, which is the substrate for Raldh2. Raldh2 is not upregulated in the neurons, but rather in the NG2 + cells that invade the lesioned area after SCI. RA is metabolized from the retinal and secreted in association with exosomes. These are taken up by adjacent axons and serve as an important axonal/neurite growth cue.

To consolidate the hypothesis of an extracellular vesicle associated RA transfer between glial cells and neurons, we next assessed the RA content in exosomes released from NG2 + cells, astrocytes and neurons. Each culture was treated with vehicle, CD2019, retinol or retinal for 3 days and exosomes were isolated, as previously described (Goncalves et al., 2015). First, to obtain confirmation of exosome isolation, these were imaged using electron microscopy (EM), representative images of an exosome pool isolated from vehicle treated NG2 + cell cultures is shown in Fig. 5a. Further proof of exosome extraction was obtained by Western Blotting using the exosome inclusion marker Alix (Baietti et al., 2012) and the exclusion endoplasmic reticulum marker calnexin (Haraszti et al., 2016, Thery et al., 2006) (Fig. 5b). Next, to quantify the RA content of the various exosomes pools from neurons, NG2 and astrocyte cultures, these exosomes pools were added on F9-RARE-lacZ RA reporter cells for 24 h followed by detection of β-galactoside activity as previously described (Sonneveld et al., 1999). We found that RA was only present in exosomes from NG2 + cells and astrocytes treated with retinal, the latter containing significantly less than the NG2 + cells derived exosomes (one-way ANOVA, *p* ≤ 0.001), suggesting that they are the main source of endogenous RA (Fig. 5c, d). Since RA could also have been freely released, we repeated the assay using conditioned media from vehicle and retinal treated NG2 + cells after exosome isolation. We found that the RA levels in both were barely detectable (Fig. 5e, f). Next, to directly link NG2 + cells secreted exosomes containing RA to neurite outgrowth we measured neurite length in neurons cultured with exosomes from retinal or vehicle treated NG2 + cells. As anticipated, there was a significant increase (20%, *p* ≤ 0.001) in neurite length in the former (Fig. 5g). Taken together, these results suggest a model where neuronal pro-regeneration intrinsic mechanisms stimulated by RARβ activation (Goncalves et al., 2015) lead to an upregulation of ADH7 and synthesis of retinal. This is likely released extracellularly and taken up by the adjacent NG2 + cells that populate the injured area. Consequently, Raldh2 is upregulated in the NG2 + cells and RA synthesis increases. RA is then secreted in exosomes, either as a cargo or in association with the membrane, which are taken up by the adjacent neuronal tips where a high concentration of RARβ is found. This depicts a novel mechanism of interplay between neurons and NG2 + glia whereby synthesis and transfer of RA is controlled by the activity of both cell types who in synergy promote axonal growth and guidance (Fig. 6).

## 3 Discussion

In this study, we show that (1) one of the signalling cascades induced by RARβ activation in the injured neurons is endogenous RA signalling in NG2 + cells and neurons at the injury site, (2) the RA paracrine signalling thereof initiated has an important effect in axonal outgrowth and (3) is controlled by the release of exosomes from NG2 + cells.

### 3.1 Synthesis of RA is essential for the NG2 + cells to form a “glial sling” for axonal growth

During development RA acts as axon/neurite guidance cues (Dmetrichuk et al., 2006, Farrar et al., 2009, Maden et al., 1998), and here we show that this effect can be recapitulated in the adult where RA is essential to guide the growing axon through the inhibitory DREZ in the mature CNS. Loss of function of Raldh2 results in the absence of the PNS-CNS axonal bundles that are otherwise abundant in our avulsion regeneration model. The NG2 + cells, where the last step in the RA synthesis occurs via Raldh2, are the major source of RA in this system. The NG2 + cell release of RA acts as the attractant signal, and could be a major determinant factor in the anatomical arrangement of the “glial sling” described during development and in adult regenerating systems (Raper and Mason, 2010).

The NG2 + cells that invade the sites after SCI (Fidler et al., 1999, Jones et al., 2002, Tang et al., 2003, Zhang et al., 2001); albeit having been reported to express Raldh2 for a short period after the injury (Mey et al., 2005), have been largely perceived as detrimental to regeneration. This is likely to reflect an attempt to carry out appropriate axonal guidance but in the absence of growing axons, the phenotype of the NG2 + cells as potential RA sources cannot be sustained. Our results suggest that the retinal synthesized by the regenerating axons may act as the physiological signal of growth to the NG2 + cells and provide a positive feedback in a retinal-RA transfer between the two cells while guidance through the CNS is required. The specific distribution of the ADH7 and Raldh2 is therefore paramount in this assembly.

### 3.2 Exosomal transfer of RA fine tunes cellular effects

Due to its importance across numerous cell functions, RA signalling is subject to tight regulation with the differential and often dynamic expression patterns of specific synthesizing and metabolising enzymes accounting for the precise control of RA distribution within embryonic cell populations (Rhinn and Dolle, 2012). Here we identify a novel mode of RA signalling in the adult that reflects an equally strict control. The transfer of RA in association with exosomes between NG2 + cells and neurons suggests that a precise distribution of RA is required to elicit the appropriate biological effect. The association with exosomes may allow a certain concentration of RA be specifically delivered to the desired target. Simple diffusion would not be able to confer cell specific control for the uptake of RA nor for the concentrations delivered.

### 3.3 Novel role for NG2 + cells in axonal regeneration

The mechanisms described herein shed new lights to the neuron-glial cross-talk that has been recently emphasised specifically with regard to astrocytes, as a key element in the successful regeneration of the adult CNS (Anderson et al., 2016). We extend this concept to the NG2 + cells and show that an endogenous signalling pathway that is known to play an important role in directing path guidance and orphan partition during development of the CNS and PNS (Maden, 1998, Maden et al., 1996), can be recapitulated in the adult in response to neuronal RARβ activation after SCI. Thus, the re-awakening of a developmental signalling system may be crucial for repair in the adult and as such, our study suggests that RARβ, and the subsequent modulation of RA synthesis at the injury site could represent important therapeutic targets for CNS regeneration.

### 3.4 Therapeutic implications

The identification of an autonomous mechanism that perpetuates axonal regeneration is of utmost importance in translational medicine as the challenge in human spinal cord regeneration is the long distance the axons need to grow (Chew et al., 2012) thus requiring lengthy treatments. Our data provide the possibility of an advantageous therapeutic approach whereby an RARβ agonist could be administered for a much shorter period than the one required for the full regeneration of the axon, as the endogenous RA signalling would maintain the required axonal growth thereafter.

## 4 Materials and methods

### 4.1 Surgery, lentiviral transduction and drug treatments

All rat experiments were approved by the local veterinarian and ethical committees and carried out according with the UK Home Office regulations. In male Sprague-Dawley rats (8 per treatment group for each set of experiments) C5–C8 and T1 dorsal roots were cut flush with the SC surface. The cut ends of the dorsal roots were subsequently introduced through slits in the pia mater and positioned superficially in the SC adjacent to where they had been cut.

For Raldh2 loss of function studies, 5 μl of lentivirus (titer: 3.67 × 10^8^ TU/ml) was injected manually at the DREZ of the severed sensory roots, using a 20 μl Hamilton syringe at 0.5 μl min^− 1^ and the needle was left in place for a following 5 min to limit diffusion through the needle tract. Lentivirus were provided by GeneCopoeia. The shRNA to rat Raldh-2, sequence gatccgggcatagacaagattgcattctcaagaggaatgcaatcttgtctatgccttttttg was ligated into psi-LVRH1GH. The lentiviral particles were generated by following a standardized protocol using highly purified plasmids and EndoFectin-Lenti™ and TiterBoost™ reagents. The lentiviral transfer vector was co-transfected into 293Ta cells with Lenti-Pac™ HIV packaging mix.

Rats were treated with: vehicle or a specific RARβ gonist, CD2019 (synthesized by Sygnature Chemical Services, Nottingham, UK, 1 mg/kg), by intraperitoneal injections (ip), three times a week for 4 weeks. Animals were culled after 4 weeks of treatment. Rats were perfused transcardially with heparinised 0.9% NaCl solution and 4% paraformaldehyde in 0.1 M phosphate buffer. The cervical cords with attached DRGs were dissected, rapidly removed and post-fixed with 4% paraformaldehyde (in 0.1 M phosphate buffer) for at least 2 days at room temperature. Tissue was then embedded in paraffin wax and 5 μm longitudinal or transversal sections cut throughout each block. Sets of consecutive sections, comprising the lesioned area, were used for immunostaining.

### 4.2 Immunohistochemistry and antibodies

Immunohistochemistry was carried out as previously described (Goncalves et al., 2005).

Antibodies used were: mouse monoclonal anti-βIII tubulin (Promega, 1:1000); chicken polyclonal anti-GFAP (Abcam, 1:300); rabbit polyclonal anti-GFAP (DAKO, 1:2500); mouse monoclonal anti-GFAP (Sigma, 1:100); rabbit polyclonal anti-RARβ (Santa Cruz Biotechnology, Inc., 1:100); Rabbit polyclonal anti-NG2 (Millipore, 1:100); goat polyclonal anti-aldehyde dehydrogenase1A2 (Raldh2) (Santa Cruz Biotechnology, Inc., 1:100); goat polyclonal anti-alcohol dehydrogenase 7 (class IV) (ADH7) (Santa Cruz Biotechnology, Inc., 1:50); chicken polyclonal anti-microtubule associated protein 2 (MAP2) (Abcam, 1:1000); mouse monoclonal AC15 anti-beta actin (1:10,000, Sigma-Aldrich), rabbit polyclonal anti-AIP1/Alix (1:1000 for western blotting and 1:100 for immunocytochemistry, Millipore); anti-Calnexin antibody (1:1000 Abcam); chicken polyclonal anti-GFP antibody (Abcam, 1:500). Secondary antibodies for immunohistochemistry were AlexaFluor™ 594, AlexaFluor™ 488 and AlexaFluor™ 647 (1:1000, Molecular Probes, Life Technologies). DAPI was used to stain nuclei (1 μg/mL, Sigma Aldrich).

### 4.3 Cell culture treatments

Cell cultures were treated as described with:

Dimethylsulphoxide (DMSO) was used as vehicle, and the retinoids were used in × 1000 stock concentrations in DMSO. The retinoids used have been previously described (Goncalves et al., 2009). They were: a RARβ selective agonist (CD2019), retinol and retinal and were all synthesized by Sygnature Chemical Services (Nottingham, UK) and used at 10^− 7^ M. Other culture treatments were: the Raldh2 inhibitor DEAB (4-diethylaminobenzaldehyde) from Aldrich at 10^− 4^ M, the pan RAR antagonist BMS189453 synthesized by Sygnature Chemical Services at 10^− 7^ M, GW4869 at 10^− 6^ M. NG2 + cell cultures were also treated with exosomes extracted from neuronal cultures that had been treated either with vehicle or retinal (exosomes extracted from one well of a 6 well plate were added to a chamber of NG2 + cells cultured on a 8 well chamber). Culture conditions were three wells per treatment carried out three times.

### 4.4 Confocal microscopy

Multichannel fluorescence (DAPI–FITC–Texas Red filter set) images were captured using a Zeiss LSM 700 laser-scanning confocal microscope, with a 63 x oil-immersion Aprochromat objective (Carl Zeiss). Settings for gain, aperture, contrast and brightness were optimized initially, and held constant throughout each study so that all sections were digitized under the same conditions of illumination. Channels were imaged sequentially to eliminate bleed-through and multichannel image overlays were obtained using Adobe Photoshop 7.0 (Adobe Systems). For time-lapse imaging analysis, cells were imaged every 15 min for 24 h while at 37 °C and 5% CO_2_. The Axiovision software was used to collect information on pixel immunoreactivity used for quantitative purposes.

### 4.5 Electron microscopy

Aliquots of 2.5 μl of exosomes were placed on Formvar coated grids and allowed to settle for 1–2 min without being allowed to dry. Exosomes were fixed with 2% glutaraldehyde for 5 min, washed three times with distilled de-ionised water then contrasted with 1 part 3% uranyl acetate in 9 parts 2% methyl cellulose for 10 min. Visualisation of exosomes was by a FEI Tecnai T12 BioTWIN transmission electron microscope fitted with an AMT camera.

### 4.6 Quantifications of immunofluorescent staining in the spinal cord

Quantitative analysis of Raldh2 and ADH7 was carried out as previously described (Herrmann et al., 2010). In brief, positively stained areas were quantified as the pixels of immunoreactivity above a threshold level per unit area. The threshold value was set to include fluorescent positive signal and to exclude background staining. Threshold values for a given section and stain remained the same throughout the study. The number of pixels was measured in a 300 μm^2^ area comprising the DREZ of the implanted severed dorsal roots and the contiguous spinal cord. At least ten sections per rat were used for these quantifications and the operator was blinded to the treatments.

To quantify axonal stumps at the DREZ (area of 300 μm^2^ in PNS-CNS interface), the number of dystrophic growth cones were manually counted in high power images of the DREZ area, by an observer blinded to the treatments. At least 10 sections per rat from 4 rats were used for these quantifications.

### 4.7 Neurite outgrowth assay

Outgrowth in the cell cultures was carried out as previously (Goncalves et al., 2005).For neurite outgrowth assay, the neurons were stained with anti-βIII tubulin antibody (Promega, 1:1000) followed by anti-mouse IgG-Alexa594 or 488, and DAPI was used to stain nuclei (1 mg/ml, Sigma Aldrich, Dorset, UK). Six random images of 40mm^2^ in each well were captured on an InCell Analyser and the images were imported into an InCell analyser Developer Toolbox (GE Healthcare) image analysis software package whereby mean total neurite length was calculated in triplicate wells per experiment. Results were repeated three times independently.

### 4.8 F9-RARE LacZ reporter assay

Murine F9 embryonal carcinoma cells which stably express an RARβ2-promoter construct were used as previously described (Sonneveld et al., 1999). Cells were scored on a blue vs. not blue basis and number or percentage of F9-RARE LacZ-positive cells were quantified from five random fields of view from three independent experiments (Sonneveld et al., 1999).

### 4.9 Primary neuronal cell cultures

Mouse primary cortical neurons were prepared as previously described (Goncalves et al., 2013).

### 4.10 NG2 + cell culture

Primary mixed glial cultures were prepared as described previously (Goncalves et al., 2013) using a modified protocol. Briefly, mixed glial cultures were obtained from the cortices of postnatal mice (P5). Cultures were maintained at 37 °C (5% CO_2_/95% O_2_) in DMEM/F12 medium supplemented with 15% fetal bovine serum (FBS) (Invitrogen, Life Technologies), 9 mM glucose (Sigma Aldrich), 2 mM l-glutamine (Invitrogen) and 1% penicillin-streptomycin (Invitrogen) on poly-d-lysine (PDL, 5 μg/ml, Sigma-Aldrich) pre-coated 75 cm^2^ flasks for 10 days. Microglial cells were then harvested by forcefully tapping at the side of the flasks for 6 times. Medium was collected and centrifuged at 1000 rpm for 5 min. The cells were plated in a 75 cm^2^ flask at 37 °C for 30 min to allow microglial cells to attach. Medium was collected again and centrifuged at 1000 rpm for 5 min. Cells were re-suspended in NG2 growth medium [DMEM/F12 supplemented with 1% N2, 2% B27, 2 mM l-glutamine, 1% penicillin-streptomycin, 10 ng/ml FGF2 (R&D Systems) and 10 ng/ml PDGF-AA (Cell Guidance Systems)] and plated in PDL pre-coated culture vessels at the density of 30,000 cells/cm^2^ for NG2-neuron co-culture. The purity of the NG2 cultures was confirmed by routine immunostaining with ~ 95% of the cells being NG2 positive. For the F9-RARE LacZ reporter assay, 99% confluent NG2 cells were cultured in PLD pre-coated 75 cm^2^ Thermo Scientific™ Nunc™ Cell Culture flasks with Filter Caps for 3 days as described.

### 4.11 NG2 + cells-neuron co-culture

NG2 + cells were harvested as mentioned above and plated in PDL pre-coated culture vessels at the density of 30,000 cells/cm^2^. The next day, 4000 neurons were added into the culture. Cells were then treated for 3 days as described.

### 4.12 Exosome isolation

Exosomes were prepared from conditioned media from cell cultures, using Total Exosome Isolation (TEI) reagent (for cell culture media) from Invitrogen, Life Technologies, in accordance with the manufacturer's instructions. Conditioned media were centrifuged at 2000*g* for 30 min, 4 °C to remove cells and cell debris and the resulting supernatants were mixed with 0.5 volumes of TEI reagent and centrifuged at 10,000*g*, 4 °C, 1 h, following overnight 4 °C incubation. Exosome pellets were twice washed by re-suspension in ice-cold PBS followed by centrifugation at 100,000*g*, 1 h, 4 °C (Gardiner et al., 2016). Unused intact exosomes were stored at − 80 °C as PBS suspensions. For F9-RARE-lacZ RA reporter cells experiments, a pool of exosomes isolated from a 75 cm^2^ Thermo Scientific™ Nunc™ Cell Culture treated flasks with Filter Caps of 99% confluent mouse cortical neurons, or astrocytes or NG2 + cells were used.

### 4.13 Western blotting

Proteins were separated by SDS-PAGE on 10% (w/v) polyacrylamide gels and then transferred to a 0.45 μm pore size nitrocellulose membrane (BA85; Schleicher and Schuell) using a Trans-Blot SD Semi-Dry Transfer Cell (Bio-Rad Laboratories) Nitrocellulose membranes were incubated in blocking solution consisting of 5% (w/v) skimmed milk powder in PBS-Tween-20 (0.1%, w/v) (PBS-T) for 1 h at room temperature, followed by incubation with the appropriate primary antibody diluted in blocking solution overnight at 4 °C. Membranes were washed in PBS-T and then incubated with species-specific secondary antibodies in blocking solution for 1 h at room temperature in the dark, after which time the membranes were washed as above. Protein levels were corrected for loading differences by normalizing against β-actin levels. Proteins were detected by scanning at 700 and 800 nm using the Odyssey detection system (LI-COR Biosciences).

### 4.14 Data analysis

Data were analysed using either Student's *t*-test one-way ANOVA followed by Tukey's or Fisher's test, using Sigma Stat software (SPSS Software Ltd., Birmingham, UK). Comparisons were made between appropriate groups and differences were considered statistically significant at the level of *p* < 0.05. Results are given as mean ± SEM and *p*-values are provided as summary statistics.
